# Coffee cysteine proteinases and related inhibitors with high expression during grain maturation and germination

**DOI:** 10.1186/1471-2229-12-31

**Published:** 2012-03-01

**Authors:** Maud Lepelley, Mohamed Ben Amor, Nelly Martineau, Gerald Cheminade, Victoria Caillet, James McCarthy

**Affiliations:** 1 Nestle Research and Development Center, 101 Avenue Gustave Eiffel, Tours 37097, France; 2 ROYAL SAT, Hacienda la Jarilla Apdo 47, 41300 San José de la Rinconada, Sevilla, Spain

**Keywords:** Cysteine proteinase, Cysteine proteinase inhibitor, Proteinase activity, Coffee

## Abstract

**Background:**

Cysteine proteinases perform multiple functions in seeds, including participation in remodelling polypeptides and recycling amino acids during maturation and germination. Currently, few details exist concerning these genes and proteins in coffee. Furthermore, there is limited information on the cysteine proteinase inhibitors which influence the activities of these proteinases.

**Results:**

Two cysteine proteinase (CP) and four cysteine proteinase inhibitor (CPI) gene sequences have been identified in coffee with significant expression during the maturation and germination of coffee grain. Detailed expression analysis of the cysteine proteinase genes CcCP1 and CcCP4 in Robusta using quantitative RT-PCR showed that these transcripts accumulate primarily during grain maturation and germination/post germination. The corresponding proteins were expressed in *E. coli *and purified, but only one, CcCP4, which has a KDDL/KDEL C-terminal sequence, was found to be active after a short acid treatment. QRT-PCR expression analysis of the four cysteine proteinase inhibitor genes in Robusta showed that CcCPI-1 is primarily expressed in developing and germinating grain and CcCPI-4 is very highly expressed during the late post germination period, as well as in mature, but not immature leaves. Transcripts corresponding to CcCPI-2 and CcCPI-3 were detected in most tissues examined at relatively similar, but generally low levels.

**Conclusions:**

Several cysteine proteinase and cysteine proteinase inhibitor genes with strong, relatively specific expression during coffee grain maturation and germination are presented. The temporal expression of the CcCP1 gene suggests it is involved in modifying proteins during late grain maturation and germination. The expression pattern of CcCP4, and its close identity with KDEL containing CP proteins, implies this proteinase may play a role in protein and/or cell remodelling during late grain germination, and that it is likely to play a strong role in the programmed cell death associated with post-germination of the coffee grain. Expression analysis of the cysteine proteinase inhibitor genes suggests that CcCPI-1 could primarily be involved in modulating the activity of grain CP activity; while CcCPI-4 may play roles modulating grain CP activity and in the protection of the young coffee seedlings from insects and pathogens. CcCPI-2 and CcCPI-3, having lower and more widespread expression, could be more general "house-keeping" CPI genes.

## Background

Cysteine proteinases (CP) represent a large group of proteins in plants, with over 140 annotated gene sequences identified to date in the Arabidopsis genome [1-3]. As expected for such a large family, the functions of these proteins are diverse, ranging from involvement in programmed cell death (PCD) [4,5] to influencing tissue development [6,7] and pathogen response signalling [8,9]. During seed development, cysteine proteinases have been found to participate in PCD events associated with embryogenesis [10] and seed coat formation [11], as well as playing a role in the processing of proteins, particularly the seed storage proteins found in protein storage vacuoles [12]. Different cysteine proteinases are also thought to make a major contribution to the mobilization of the stored seed protein reserves as germination progresses [13,14]. In germinating mung bean seeds, it has been shown that at least two cysteine proteinases are induced soon after germination has started [15], and these authors proposed that vacuolar receptors (VCRs) transport these newly made proteinases to the protein storage vesicles (PSVs) thereby enabling them to participate in the mobilization of the seed protein reserves.

In plants, protein hydrolysis via cysteine proteinases is thought to be modulated, at least in part, by a group of proteins called the cysteine proteinase inhibitors. These polypeptides, also called phytocystatins, are a group of plant polypeptides that inhibit C1A and C13 type plant cysteine proteinases by acting as pseudosubstrates [16,17]. While it is believed that the key biological function of the plant cysteine proteinase inhibitors (CPI) is to modulate the function of target proteinases *in-vivo*, to date, only a limited number of CPI have been tested with plant cysteine proteinases. In one such study [14], the inhibitory effects of a series of recombinant barley CPI were tested against multiple barley cathepsin L-like cysteine proteinases. These authors showed that most of the barley CPIs showed activity against all the CP's tested, although a few CPI did show increased inhibition effects towards one or two specific barley cysteine proteinases. CPIs have attracted particular attention due to their capability to inhibit cysteine proteinases found in the digestive tracts of herbivorous insects, an effect that can significantly reduce the destructive effects of these insects [18,19]. For example, Urwin et al. [20] showed that over-expression of sunflower or rice CPI polypeptides in potato increased its resistance to *Globodera *root nematodes, and it has been demonstrated that simultaneously over-expressing a CPI with a second protease inhibitor acting on another protease family (carboxypeptidases) allowed tomato plants to have protection for a longer duration from two different tomato pathogens due to a reduced build-up of insect tolerance [21]. Plant CPIs have been also been demonstrated to increase tolerance to fungal and bacterial pathogens in transgenic plants [22].

Coffee is one of the most important agricultural commodities traded worldwide, however, there continues to be a lack of fundamental knowledge on many aspects of this crop. To date, for example, there is little information on the proteinase and proteinase inhibitor genes of coffee. As shown above, the cysteine proteinases and their inhibitors play important roles in plant seeds. Thus, we decided to begin an investigation of the CP/CPI genes expressed in the semi-recalcitrant coffee grain. In addition, because amino acids and peptides are an important group of coffee flavour/aroma precursors in coffee [23,24], such a study could also yield some clues regarding the potential role of CP/CPI gene products on coffee quality. In this work, we describe cDNA representing several coffee CP and CPI genes, and we present the expression of these genes in developing and germinating grain. To begin studying the functional properties of two highly expressed CP proteins, we have also expressed these proteins in *E. coli *and tested the recombinant polypeptides for protease activity. The results obtained are discussed in relation to the potential roles of the gene products in the development and germination of the coffee grain.

## Methods

### Plant material

#### Robusta samples

The *Coffea canephora *(BP409) "maturation" tissues (roots, branches, leaves and cherries at different stages of development) were harvested in 2007 from field grown trees (Equator), immediately put into liquid nitrogen, then held at -20°C before being sent frozen to Tours, France. Once at Tours, these samples were kept at -80°C until use.

Coffee cherries of *Coffea canephora *(BP409) used to obtain the "germination" tissues were harvested at mature stage from field grown trees in Equator in 2008, and sent to Tours at room temperature. On arrival, they were manually depulped, washed, and the light grain removed by floating. The remaining grain were dried and the tegument were manually removed. Subsequently, the grain were sterilized by a 1 h treatment in calcium hypochlorite (50 g/l), followed by three washes using sterile water. The grain were then incubated *in vitro *on Heller medium without added sugar or hormone (Agar 7 g/l), at room temperature (25°C). Then five grain were harvested at various times (DAI = Days After Imbibition), and frozen in liquid nitrogen. In the experiment presented, 14DAI (T4) corresponds to the first sign of radical emergence.

*Coffea arabica *(T2308) leaves were harvested at different stages of development, in 2006, from trees grown under greenhouse conditions at Tours, France and kept at -80°C before use. Two independent sets of leaves were harvested. The development stages of the leaves are defined as follows: Very Young Leaves (VYL), Young Leaves (YL), Mature leaves (ML), Old Leaves (OL). The sizes of the leaves collected were: approximately 2-3 cm for VYL stage; 6-9 cm for YL stage and 12-15 cm for ML and OL stages.

### RNA preparation

The samples from the various tissues were reduced to a powder in a SPEX CertiPrep 6800 Freezer Mill with liquid nitrogen and the powders were then stored at -80°C until total RNA was extracted. In the case of the coffee cherries at different stages of development, these were first separated into pericarp and grain tissues and then each was very rapidly reduced to a powder and stored as described above. RNA were extracted and purified from the stored powders using the RNeasy Plant mini kit (QIAGEN) that included a DNase treatment using the manufacturer's instructions. The quality of the final RNA samples obtained were checked by agarose gel electrophoresis and ethidium bromide staining.

### cDNA synthesis

The method used to make the cDNA was very similar to the protocol described in the Transcriptor Reverse Transcriptase kit (Roche) using around 1 μg total RNA sample and 870 ng of oligo dT(18) (Proligo), with reactions performed at 55°C for 30 min. The cDNA samples generated were then diluted one hundred fold in sterilized water and aliquots were stored at -20°C for later use in QPCR experiments.

### DNA sequence analysis

Plasmid DNA was purified using Qiagen kits according to the instructions given by the manufacturer. Prepared plasmid DNA was then sequenced by the dideoxy termination method [25]. Computer analyses were performed using the Laser Gene software package (DNASTAR, version 7.1.0).

### Real time QRT-PCR experiments

cDNA prepared as described above was employed for the quantitative RT-PCR experiments using TaqMan probes, as described by Simkin et al. [26], with an Applied 7500 instrument; except the cDNA dilutions and the Taqman primers/probes were different. The QRT-PCR primers and TaqMan probes were designed using Primer Express^® ^software v2.0 from Applied Biosystems and are listed in Table 1, below.

**Table 1:** Primer and TAQMAN probes used for the quantitative PCR experiments.

Primers and Probes	Sequences
rpl39-F1	^5'^GAACAGGCCCATCCCTTATTG^3'^

rpl39-R1	^5'^CGGCGCTTGGCATTGTA^3'^

**rpl39-MGB1**	**^5'^ATGCGCACTGACAACA^3'^**

CP1-F1	^5'^ACACAGACCTCTTGATACCAAAACAT^3'^

CP1-R1	^5'^TCTTCCAAGAGCAAACCACCTT^3'^

**CP1-MGB1**	**^5'^TCTGCTCTTCAGAGGTTGTA^3'^**

CP4-F1	^5'^CAGGATGCAACGTGGTGTTG^3'^

CP4-R1	^5'^CCTCCATTGCTATCCCACAAA^3'^

**CP4-MGB1**	**^5'^TGCTGCTGAAGGCG^3'^**

CPI1-F1	^5'^TGTTTGGGAGATCTAATCTGATGATT^3'^

CPI1-R1	^5'^AAACCGAACACTTAACAGCAAAGA^3'^

**CPI1-MGB1**	**^5'^TTAGTACCTTTCAGTGCAAAT^3'^**

CPI2-F1	^5'^CGCTATTGCCTATTTGCCTAGTAGA^3'^

CPI2-R1	^5'^GAAACTCCAATCTTTCCAACTGAAA^3'^

**CPI2-MGB1**	**^5'^TAAAGCTAACGCGTAAATG^3'^**

CPI3-F1	^5'^AACCGACGCTGCAAGAATG^3'^

CPI3-R1	^5'^CAGGGTGGTGAGTAGGAGGAGAT^3'^

**CPI3-MGB1**	**^5'^CTTCTGCCTTTCCC^3'^**

CPI4-F1	^5'^TGTTTATGGTGTGGCTTTCAGTTT^3'^

CPI4-R1	^5'^CGTAGGGAGACGTATGCATGAC^3'^

**CPI4-MGB1**	**^5'^TGCATGGATGATGTACTG^3'^**

All MGB Probes were labeled at the 5' end with the fluorescent reporter dye 6-carboxyfluorescein (FAM) and at the 3' end with the quencher dye 6-carboxy-tetramethyl-rhodamine (TAMRA), except rpl39 probe which was labeled at the 5' end with the fluorescent reporter dye VIC and at the 3' end with quencher TAMRA

Quantification was carried out by the method of relative quantification, using the constitutively expressed ribosomal protein rpl39 as the reference. In order to use the method of relative quantification, it was necessary to show that the amplification efficiency for the different gene sequences were roughly equivalent to the amplification efficiency of the reference sequence (rpl39 cDNA sequence) using each specifically defined primer and probe sets. To determine this relative equivalence, plasmid DNA containing the appropriate cDNA sequences were diluted 1/1000, 1/10,000, 1/100,000, and 1/1,000,000 fold, and using the QPCR conditions described above, the efficiencies of amplification were calculated. All the primer/probe sets showed acceptable efficiencies.

### Production of recombinant *Coffea canephora *CcCP1 and CcCP4 in *E. coli*

Expression vectors were generated using the "Champion™ pET SUMO Protein Expression System" (Invitrogen). The CcCP1 sequence minus its N-terminal 28 amino acids was amplified by PCR as follows: 50 μl reactions contained the plasmid pA4-43, 5 μL of TaKaRa^® ^DNA Polymerase 10X LA PCR^® ^Buffer, 600 μM of each CcCP1 specific primers (CP1-FP ^5'^ATGTTCCAACATGAAATTCAGTATC^3' ^and CP1-RP ^5'^TCAAGAGGTCTGTGTCACCA^3'^), 200 μM each dNTP, and 0.5 U of TaKaRa DNA Polymerase (Takara Bio Inc). The PCR cycling conditions were as follows: 94°C for 2 min; then 35 cycles of 94°C 1 min, 55°C 1.5 min, and 72°C 1.5 min followed by a final step at 72°C 7 min. The PCR product was then gel purified. The CcCP4 sequence minus its N-terminal 22 amino acids was produced as described for the CcCP1 insert except the initial DNA substrate was plasmid pcccs46w7n5, and the specific primers were (CP4-FP ^5'^ATGGAGATCACAGAAAGAGATT^3' ^and CP4-RP ^5' ^CTAGAGGTCGTCCTTAGGT^3'^).

The gel purified fragments were then cloned into the TA cloning site of the pET-SUMO vector, as recommended by the vector manufacturer. Ligated plasmids were transformed into One Shot^® ^Mach1™-T1^R ^Chemically Competent Cells (Invitrogen). Clones with the inserts in the correct orientation were selected by PCR screening and the plasmid containing CcCP1 was named pNM17 and the plasmid containing CcCP4 was named pNM6.

For protein expression, BL21(DE3) One shot^® ^Chemically Competent Cells (Invitrogen) were transformed with pNM17 and pNM6 plasmids as recommended in the manufacturer's protocol. Five ml overnight cultures of the selected transformants were used to inoculate 100 ml cultures of LB medium containing 50 μg/ml kanamycin (except control, i.e.: untransformed BL21(DE3) cells). The cells were grown at 37° and 200 rpm shaking to an OD_600 _of 0.4-0.6. Then, 90 ml was taken and "induced" by addition of IPTG (1 mM final). Both "Induced" and "Not Induced" cultures were further incubated at 37°C (200 rpm shaking) for 5.5 h, followed by centrifugation at 6000 g for 10 min at 4°C. Cell pellets were resuspended at room temperature in BugBuster^® ^Protein Extraction Reagent ((Novagen)) using 5 ml reagent per gram of wet cell paste. Then, 25 U benzonase nuclease (Novagen) and 1 KU rLysozyme solution (Novagen) were added per 1 mL Bugbuster and incubated 25 min at 70 rpm, at room temperature, followed by centrifugation at 6000 g for 30 min at 4°C.

The pellets obtained from the induced cultures, which contained the inclusion bodies, were again resuspended in BugBuster^® ^solution using the same volume that was used to resuspend the initial cell pellet (5 mL per gram of initial wet cell pellet) and 1 KU rLysozyme solution was added per 1 mL BugBuster^® ^and the mixture was incubated at room temperature for 5 min. Then, 6 volumes of 1/10 diluted BugBuster^® ^solution was added and the tubes vortexed for 1 min. The resulting suspensions were centrifuged at 5000 g for 15 min at 4°C. The "washed" inclusion bodies collected were resuspended in 7 volumes of 1/10 diluted BugBuster^® ^solution and centrifuged as previously. This wash step was repeated three more times to remove non-specific material associated with the inclusion bodies. The final pellets of the purified inclusion bodies (IBS) obtained were resuspended in 2 volumes of denaturing buffer A (8 M urea, 50 mM NaH_2_PO_4_, 10 mM Tris-HCl adjusted to pH8 with NaOH) and incubated at 28°C for 1 h as described by Zhang et al. [4], 2 volumes of buffer B (8 M urea, 50 mM NaH_2_PO_4_, 10 mM Tris-HCl adjusted to pH6.3 with HCl) were then added and purification was carried out with Ni-NTA Superflow Columns (QIAGEN). Briefly, the Ni-NTA slurry was mixed with the denatured proteins by shaking on a rotary shaker for 1 h at 70 rpm at room temperature, followed by a loading of the slurry on an empty column and collecting the flow-through. Two successive column washes were carried out with 1.2 volumes Buffer B, followed by elution of the recombinant proteins with 0.6 volumes of buffer C (8 M urea, 50 mM NaH_2_PO_4_, 10 mM Tris-HCl adjusted to pH5.9 with HCl), giving a fraction called El1. This was followed by 2 elutions of 0.6 volumes with buffer D (8 M urea, 50 mM NaH_2_PO_4_, 10 mM Tris-HCl adjusted to pH4.5 with HCl) giving fractions El2 and El3. 20 μl samples of the different fractions were analysed by SDS-PAGE gel electrophoresis and those containing recombinant proteins were pooled.

### Dialysis/Refolding method

Pooled fractions containing purified recombinant proteins were dialyzed in a similar fashion to that described by Zhang et al. [4]; briefly, the purified protein fractions were introduced into Slide-A-lyser^® ^dialysis cassettes (10 kD MWCO Pierce). Then, 4 successive dialysis steps were carried out for 3 h at 4°C with stirring using buffers containing decreasing levels of urea (6 M, then 4 M, 2 M and 0 M urea, see Table 2 below). The dialysis buffer volumes were approximately 50 times higher than the volumes of extracts.

**Table 2:** Names and composition of dialysis buffers

Names of dialysis buffers	Composition
Buffer 6 M	50 mM potassium phosphate pH10.7, 5 mM EDTA, 1 mM reduced glutathione, 0.1 mM oxidized glutathione, **6 M **urea

Buffer 4 M	50 mM potassium phosphate pH10.7, 5 mM EDTA, 1 mM reduced glutathione, 0.1 mM oxidized glutathione, **4 M **urea

Buffer 2 M	50 mM potassium phosphate pH10.7, 5 mM EDTA, 1 mM reduced glutathione, 0.1 mM oxidized glutathione, **2 M **urea

Buffer 0 M	50 mM potassium phosphate pH10.7, 5 mM EDTA, 1 mM reduced glutathione, 0.1 mM oxidized glutathione, **0 M **urea

### Assay for cysteine protease activity

The assay for cysteine protease activity used here is a slight modification of the one developed by Zhang et al. [4] and Troen et al. [27]. Protein samples made up to 10 μl with water were mixed with 20 μL 50 mM sodium formate buffer (pH3), then incubated 30 sec at 37°C for activation, with parallel "non-activated" control reactions set up with samples in which 20 μL milliQ pure water replaced the 20 μL sodium formate buffer. This was immediately followed by the addition of 6.7 μl of the "reaction mix" (1% BSA, 1xPBS and 6 mM L-cysteine, pH7.5). The enzyme reactions were subsequently incubated at 37°C and 3 μL aliquots were taken at different times and added to sample loading buffer, heated for 7 min at 95°C, then run on SDS-PAGE gels and stained with coomassie.

## Results

### Identification of cysteine proteinase sequences expressed during coffee grain maturation

Coffee cDNA encoding cysteine proteinases were found by carrying out BLAST searches against the Nestlé/Boyce Thompson Institute coffee EST database (*Coffea canephora *built #3, located at http://solgenomics.net/) [28] using the protein sequences of two biochemically characterized cysteine proteinases: NtCP56-KDEL from *Nicotiana tabacum *(genbank accession number ACB70409[4]) which is a peptidase from the C1A subfamily (MEROPS database nomenclature; http://merops.sanger.ac.uk[2]), and *SlCP *from *Solanum lycopersicum *(genbank accession number CAH56498[5]) which is from the peptidase C13 family (asparaginyl endopeptidase, cysteine catalytic type). This analysis yielded 15 candidate unigene sequences (data not shown). As our main objective was to study genes highly and specifically expressed in the maturing grain, we examined the "*in-silico" *expression profiles associated with these unigenes. Three unigenes (SGN-U613831, SGN-U613447 and SGN-U620235) were found that exhibited multiple ESTs and all were found in either grain or cherry EST libraries (data not shown). Further analysis indicated that two of the unigenes (SGN-U613447 and SGN-U620235) were probably different alleles of the same gene, thus giving only two, clearly different unigenes with high expression in the grain for further study. Plasmids potentially containing the longest sequences for each unigene were then selected from our available EST libraries and fully sequenced to confirm the "*in-silico*" unigene sequences.

In the case of Unigene SGN-U613831, the plasmid pA4-43 was selected to characterize the first *Coffea canephora *CP cDNA, which we named CcCP1; the cDNA has a 1511 bp long insert, with a 1194 bp coding sequence (CDS) encoding a protein of 397 amino acids. Analysis of the protein sequence of CcCP1, performed using SignalP 3.0 server (http://www.cbs.dtu.dk/services/SignalP/) and the "Conserved Domain Database" (http://www.ncbi.nlm.nih.gov/Structure/cdd/cdd.shtml), shows it is a member of the peptidase_C1 superfamily/peptidase_C1A subfamily (papain family, clan CA) and it appears to have at least three distinct domains, a hydrophobic N-terminal signal peptide, followed by a predicted 56 amino acid long I29 inhibitor propeptide domain (with an ERFN(V/I)N like domain sequence) and a peptidase domain containing the catalytic triad of Cys, His, and Asn, as well as an active site Gln residue. The second coffee CP cDNA to be studied in detail has a 1365 bp long insert encoding a protein of 359 amino acids which we have called CcCP4 (pcccs46w7n5). The protein sequence indicates that CcCP4 is also a member of the C1 peptidase superfamily (C1A subfamily) and has the three distinct domains already described for CP1, plus an obvious ERFNIN like sequence which is located within the 55 AA long predicted I29 inhibitor domain.

**Figure 1:**
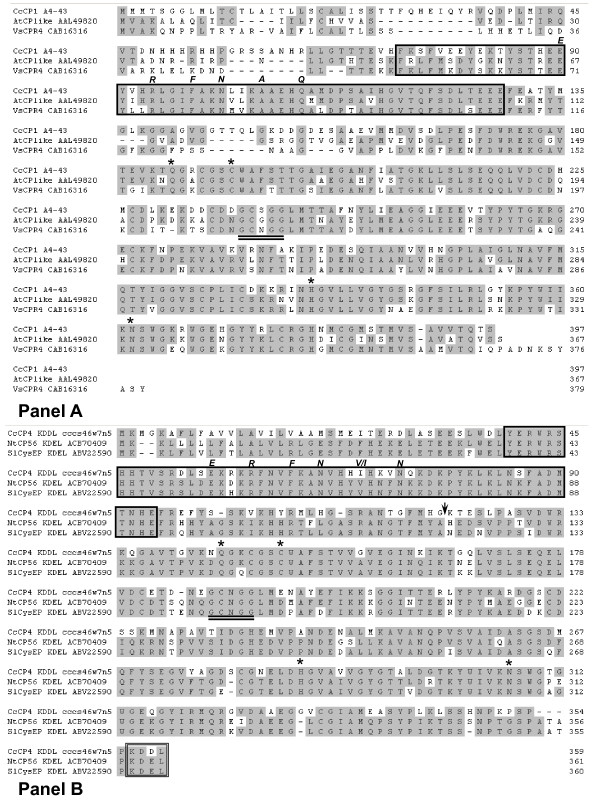
**Alignment of *C. canephora *CP1 and CP4 sequences with highly homologous plant sequences**. The alignments were done using CLUSTAL W. The key conserved amino acid characteristics and motifs are noted. Amino acids shaded in grey indicate most conserved sequences. **Panel A: **Database accession numbers are *Coffea canephora *CP1 (GeneBank sequence #AEQ54770), *Arabidopsis thaliana *AtCP (AAL49820) and V*icia sativa *VsCPR4 (CAB16316). The catalytic triad Cys, His, and Asn and also the Gln active site residues are indicated by an asterisk. The GCXGG motif is double underlined. The cathepsin propeptide inhibitor domain (I29) is shown in a rectangular box. Note: an ERFNIN-like sequence, shown in italics, exists in the propeptide region of CP1 (ie. ERFNAQ). **Panel B: **Database accession numbers: *Coffea canephora *CP4-KDDL (GeneBank sequence #AEQ54771), *Nicotiana tabacum *NtCP56-KDEL (ACB70409) and *Solanum lycopersicum *SlCysEP-KDEL (ABV22590). Symbols for key amino acids and motifs are as above, plus the ERFNIN motif is indicated in italics and the KDEL (K358-L361 for NtCP56) motif is shown in a double lined rectangular box. Arrows indicates the site of auto-hydrolysis of NtCP56 reported by Zhang et al. (2009) [4].

Figure 1A shows an alignment of the CcCP1 protein sequence with two very closely related cysteine proteinase sequences: VsCPR4 (CPR4 from *Vicia sativa *(CAB16316)) that is thought to be involved in storage protein mobilization [29] and AtCP (a putative CP protein from *Arabidopsis thaliana *(AAL49820)). Figure 1B shows the CcCP4 protein aligned with two highly related proteins; NtCP56-KDEL (*Nicotiana tabacum; *ACB70409) and SlCysEP-KDEL (*Solanum lycopersicum*; ABV22590). Zhang et al. [4] cloned a cDNA encoding NtCP56-KDEL from tobacco anthers and showed the recombinant protein produced in *E.coli *had cysteine proteinase activity after Ni-NTA purification under denaturing conditions, followed by refolding and acid activation. These authors proposed that NtCP56-KDEL was involved in pollen grain development because engineering transgenic plants to reduce expression of this gene led to plant sterility. The related SlCysEP was studied by Senatore et al. [5] and was shown to be expressed in anthers and was proposed to be associated with PCD (programmed cell death) during anther development in tomato. Figures 1A and 1B also indicate that both CcCP1 and CcCP4 possess key sequence elements associated with the C1A peptidase family; for example, the four catalytic residues Gln, Cys, His and Asn cited above plus the cathepsin propeptide inhibitor domain I29.

### Quantitative gene expression analysis for CP1 and CP4 in different coffee tissues and during grain development/germination

**Figure 2:**
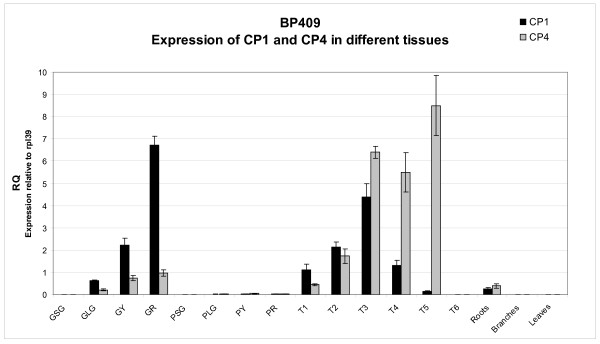
**Quantitative expression analysis of CcCP1 and CcCP4 in different tissues and at different stages for Robusta BP409**. The expression of each gene was measured in the various maturing and germinating grain samples, as well as maturing pericarp samples plus in roots, branches, and leaves. For germination stages T5 and T6, only the grain (T5) or grain/emerging cotyledons were extracted for RNA. The expression levels were obtained using quantitative RT-PCR. RQ is the expression level of each gene relative to the constitutively expressed gene RPL39. GSG, small green stage grain; GLG, large green stage grain; GY, yellow stage grain; GR, red stage grain. PSG, pericarp small green stage; PLG, pericarp large green stage; PY, pericarp yellow stage; PR, pericarp red stage. T1 is the sterilized and washed material obtained just before placing on the "germination" media; T2 = 3 DAI, T3 = 5 DAI, T4 = 14 DAI (FE), T5 = FE + 1 month, T6 = FE + 2 months. DAI represents "Days After Imbibition", FE represents "First Evidence" of germination.

The tissue and development specific expression of the coffee CP1 and CP4 genes was measured using TaqMan QRT-PCR. The expression results obtained for Robusta variety BP409 (Figure 2) indicate that both genes are expressed at increasingly higher levels as grain development progresses from large green grain stage to the mature red stage, with CP1 > CP4 (RQ = 6.73 versus RQ = 0.96 at the red grain stage). Few CP1 and CP4 transcripts were detected in Robusta pericarp, leaves, or branch samples, although low levels were clearly detected in the roots (CP4 > CP1; RQ = 0.39 versus RQ = 0.24). We also examined the expression of CP1 and CP4 in the germinating and post-germination grain. The material used for this experiment is illustrated in Additional file 1. CP1 and CP4 transcripts are found in germinating/post germination grain (Figure 2), but, in contrast to the developing grain, these genes clearly have different temporal expression patterns during these periods. First, the level of both transcripts rise from T1 (starting material; CP1 RQ = 1.1 and CP4 RQ = 0.45) to T3 (5 DAI; CP1, RQ = 4.4 and CP4, RQ = 6.4). From T3 to T5 (radial protrusion, radicle growth, and early cotyledon development), the levels of CP1 transcripts in the grain fell dramatically while the level of CP4 transcripts remained high. At T6, which is primarily the first pair of leaves (cotyledons plus remnants of the grain), little or no CP1 and CP4 transcripts were detected. Overall, these expression levels are in agreement with the previous "in-silico" expression data (data not shown).

CP1 and CP4 gene expression was also measured in an independent set of Robusta samples (data not shown). The results from this second sample set were globally in agreement with the results presented in Figure 2, with a few minor differences. For example, for the second independent set of BP409 samples covering grain development, overall CP1 expression was lower (RQ = 1.54 versus RQ = 6.73 at mature red stage) and CP4 expression was found to be higher at an earlier stage in this second grain sample set. It is likely that some of these transcript level differences result from slight differences in the precise development stages of the various samples. Comparison with a second germination sample set (from Robusta variety FRT05) showed that CP1 and CP4 expression were broadly similar, with the exception that the BP409 sample set showed increased CP1 transcript levels from T1 to T 3, but the FRT05 samples set shows the levels of CP1 transcripts are highest at T1 and then fall. Nonetheless, for both sample sets, CP1 transcripts levels are very low at T5. In both germination sample sets, CP4 transcript levels are low at T1 and rose to a maximum at T5. CP1 and CP4 transcripts were barely detected at T6 for the second sample set. Interestingly, the CP4 transcript levels were significantly higher in all the Robusta FRT05 samples examined during pre-germination/germination/post germination (including T1 to T5 samples) versus the equivalent samples of Robusta BP409.

### Production of recombinant CP1 and CP4 enzymes in E. coli: purification and activity testing

**Figure 3:**
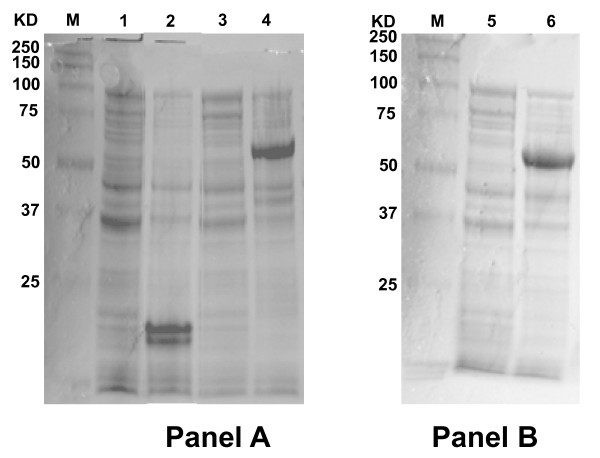
**Coomassie SDS-Page gel analysis of CcCP1 et CcCP4 recombinant proteins**. Protein samples were run on SDS-PAGE gels and stained with coomassie blue. Panel A: Lane M, molecular mass marker with the sizes shown on the left in kDa; Lane 1, empty BL21(DE3) whole cell lysate; Lane 2, Induced BL21(DE3) + pET-SUMO whole cell lysate; Lane 3, Not Induced BL21 (DE3) + pET-SUMO/CP4 whole cell lysate; Lane 4, Induced BL21 (DE3) + pET-SUMO/CP4 whole cell lysate. Panel B: Lane M, molecular mass markers with the sizes shown on the left in kDa; Lane 5, Not Induced BL21 (DE3) + pET-SUMO/CP1 whole cell lysate; Lane 6, Induced BL21 (DE3) + pET-SUMO/CP1 whole cell lysate.

In order to study the functional properties of the CcCP1 and CcCP4 proteins, we expressed these proteins, minus their respective signal peptide sequences, in *E.coli *as N-terminal HIS-SUMO fusions (see Methods for details). The results shown in Figure 3 indicate that both fusion proteins were expressed at high levels upon induction with IPTG and that their molecular weights are close to those predicted. However, subsequent analysis indicated that the vast majority of both the recombinant proteins produced were insoluble (data not shown). Therefore, we used a method similar to that described previously by Zhang et al. [4] to denature the fusion proteins and purify them by Ni-NTA affinity chromatography. The purified proteins were then re-natured as described in the methods. An SDS-PAGE analysis of samples from the different purification steps for HIS-SUMO-CP4 indicated that a significant level of purification was achieved (see Additional file 2). Similar results were obtained for the HIS-SUMO-CP1 recombinant protein.

**Figure 4:**
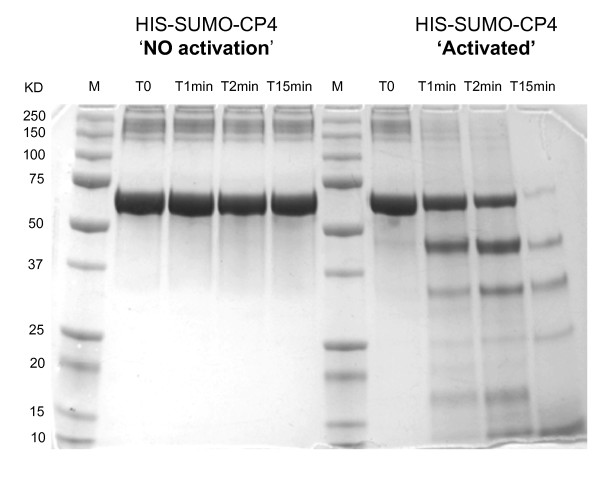
**Detection of CcCP4 protease activity**. The assay was run for different lengths of time as described in the Materials and Methods and samples were then run on SDS-PAGE gels followed by coomassie staininig. The markers (lane M) as in Figure 3. 'Activated' indicates HIS-SUMO-CP4 which has been acid treated. T = reaction times with BSA.

The purified, soluble fusion proteins were then tested for proteinase activity with a general proteinase assay based on BSA hydrolysis. In this assay, cleavage of the BSA, detected by loss of the BSA band using SDS-PAGE, indicates the presence of a proteolytic activity. In the first assays, using the full length purified HIS-SUMO-CP1 and HIS-SUMO-CP4 after refolding, no activity was detected even after long incubation times. Because both coffee proteins are in the same super family as papain, we then decided to examine the possibility that these proteinases could be activated by a short acid treatment. Figure 4 shows that the HIS-SUMO-CP4 proteinase can be activated by a short acid treatment yielding a significant level of protease activity, with the first signs of BSA degradation detected after a 1 min reaction time (clear detection of degradation products at ≈ 45 KDa and 34 KDa), and with a very pronounced degradation at T = 15 min with nearly complete disappearance of the BSA band. No BSA degradation was observed if the HIS-SUMO-CP4 polypeptide was not subjected to a low pH treatment. It was assumed that the acid treatment activates CP4 via an intrinsic autohydrolysis capability in the CP4 pro-peptide, leading to the release of an N-terminal CP inhibitor peptide as has been seen for the tobacco NtCP56 [4]. To verify that HIS-SUMO-CP4 was processed to generate a shortened, active polypeptide, the activation process was followed over time by SDS-PAGE analysis. The results obtained showed that recombinant HIS-SUMO-CP4 was processed to generate an approximately 32.6 kDa polypeptide that is presumed to be the active CP4 proteinase (Additional file 3). We confirmed that this "activated" CcCP4 fell into the cysteine proteinase class of proteases by showing that this activity was inhibited by the cysteine proteinase specific inhibitor E-64C (Additional file 4). A similar acid activation and protease test was carried out with purified and renatured CcCP1, but was not successful (data not shown). This result suggests that HIS-SUMO-CP1 may not have refolded correctly during the renaturation step. Alternatively, it is possible that the polypeptide refolded correctly, but this cysteine proteinase has another mode of activation, such as perhaps requiring the intervention of a specific proteinase to release its putative N-terminal inhibitory domain.

### Identification of cysteine proteinase inhibitors expressed during grain maturation

To find cDNA encoding coffee cysteine proteinase inhibitors, we carried out BLAST searches using the biochemically characterized *Helianthus annuus *cysteine protease inhibitor HaCPI (accession number JE0308 [30]), the *Dianthus caryophyllus *cysteine protease inhibitor DcCPIn (accession number AAK30004 [31]) and a putative cystatin-like inhibitor sequence from *Citrus × paradisi *(accession number AAG38521), as the query sequences. Using these criteria, 6 distinct unigene sequences were found (data not shown). 4 of these sequences were chosen for further study in this work. Plasmids potentially containing a complete ORF representing each unigene were isolated from the available EST libraries and fully sequenced to confirm the "in-silico" unigenes sequences. The respective gene sequences have been named CcCPI-1, CcCPI-2, CcCPI-3, and CcCPI-4. The plasmid names and the size of their inserts are presented in the Additional file 5.

### Quantitative gene expression analysis for CPI-1, CPI-2, CPI-3 and CPI-4 in different Robusta tissues and during grain development/germination

**Figure 5:**
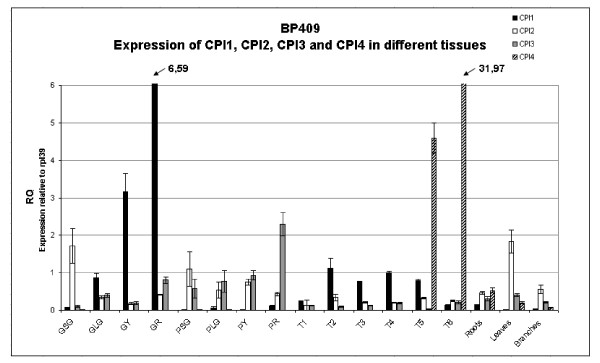
**Quantitative expression analysis of coffee cysteine proteinase inhibitor genes CPI1, CPI2, CPI3 and CPI4 in different tissues and at different grain/pericarp development stages for Robusta BP409**. The expression of each gene was measured in the various samples indicated. RQ is the expression level of each gene relative to the constitutively expressed gene RPL39 in that sample. Sample symbols are as in Figure 2.

To obtain precise information on the expression profiles of the 4 cysteine proteinase inhibitor genes in Robusta, QRT-PCR was used to measure their expression levels in a number of tissues and during grain and pericarp maturation, and during germination/post germination. The data obtained is presented in Figure 5. CPI-1 is expressed at increasingly high levels during Robusta grain development (low transcript levels in small green grain with RQ_GSG _= 0.06, but increasing transcript levels from stages large green to red with RQ_GLG _= 0.87, RQ_GY _= 3.16 and RQ_GR _= 6.59 respectively). The results obtained in an independent experiment were similar except that this second set of BP409 samples showed more constant levels of CPI-1 transcripts between the large green and red stages (after a large increase between the small green and large green stages; data not shown). In both sample sets, few CPI-1 transcripts were detected in the Robusta pericarp, leaves, and branches, although a low level was seen for one of the red pericarp samples (which appeared to be "softening") plus a low level was detected in both root sample sets. (Figure 5--red pericarp RQ_PR _= 0.12 and roots RQ_roots _= 0.13). Figure 5 also shows that CPI-1 is expressed during Robusta grain germination, starting low at T = 1 (RQ_T1 _= 0.25), then showing roughly similar transcript levels from T = 2 to T = 5 (RQ_T2 _= 1.12 to RQ_T5 _= 0.79), followed by a drop in T6 (RQ_T6 _= 0.13): in first pair of young leaves/cotyledons. Quite similar expression levels were seen in the second set of samples for Robusta grain germination (FRT05; data not shown).

Both the CPI-2 and CPI-3 genes were found to be expressed to a small extent in all the tissues examined (Figure 5). A second independent RNA set showed a similar expression profile (data not shown). Thus, these two CPI genes do not appear to have any clear tissue specificity. Detailed examination of the QRT-PCR results in Figure 5 show however that some expression differences exist for these two genes, for example there are higher CPI-2 transcript levels found at the SG grain stage (high RQ_GSG _= 1.71 for CPI-2 in Figure 5) and are slightly higher in leaves (both Robusta sample sets). One can also observe that CPI-3 transcript levels are very low at the T5 germination stage (this was also seen in the second set of Robusta samples). Another difference is the relatively high CPI-3 expression associated with the mature red coffee pericarp tissue (RQ_PR _= 2.29). Interestingly, the higher expression of CPI-3 is most noticeable in the sample used for Figure 5. As this sample could be more mature (and thus softer) than the other "mature" pericarp sample tested, it is entirely possible that increased CPI-3 expression is coupled with pericarp age and fruit softening. It will be interesting in the future to explore this possibility further by carrying out more detailed expression studies on this gene at the end of pericarp maturation, and to explore whether CPI-1 could also be involved (which also appears to rise somewhat at this stage).

The QRT-PCR results in Figure 5 show that there is little or no expression of CPI-4 in the developing grain or in the pericarp at any maturation stage examined (0 < RQs < 0.02). This was also observed with the second Robusta sample set studied (data not shown). However, Figure 5 shows that significant levels of CPI-4 transcripts occur in Robusta roots (RQ_roots _= 0.53), leaves (R_leaves _= 0.20) and branches (RQ_branches _= 0.08). In contrast however, few CPI-4 transcripts were detected in the roots and branches of the second Robusta sample (data not shown), suggesting some possible maturity or other tissue sampling related differences could be involved. In the case of leaves, relatively low CPI-4 expression was seen in the leaves of the BP409 Robusta sample set used for Figure 5, but very high levels were seen for this gene in the leaves of the BP409 Robusta sample used for the second sample set (data not shown). This expression difference, which was hypothesized to be due to leaf maturity, is explored in more detail below. Another surprising aspect of the CPI-4 expression data obtained is the extremely high level of CPI-4 expression detected during the last post-germination stages examined (Figure 5: T5 stage, RQ = 4.59 and T6 stage consisting primarily of the first two young leaves/cotyledons with RQ = 31.97). There was no significant expression of CPI-4 from germination stages T1 to T4 (14DAI, grain showing its first sign of radicle emergence). The same results were seen in the second Robusta sample set analysed (FRT05; data not shown). This suggests CPI-4 could play some important role during this period of plantlet growth/emergence.

### Quantitative gene expression analysis of the cysteine protease inhibitor genes CPI-1--CPI-4 at different stages of leaf development

**Figure 6:**
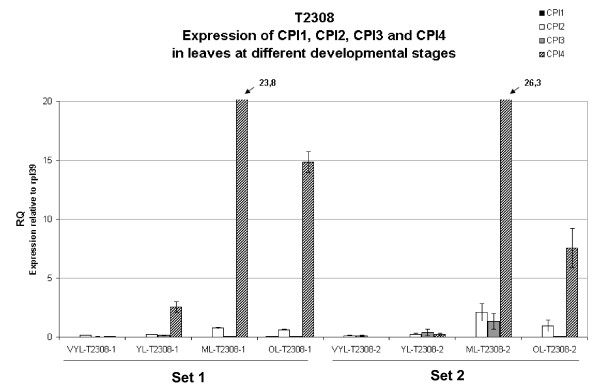
**Quantitative expression analysis of CcCPI1, CcCPI2, CcCPI3 and CcCPI4 in leaves at different maturation stages for Arabica T2308**. The expression of each gene was measured in two independent sets of leaf samples during development and senescence. The expression levels were obtained using QRT-PCR. RQ is the expression level of each gene relative to the constitutively expressed gene RPL39. Symbols: VYL, very young leaves; YL, young leaves; ML, mature leaves; OL, old leaves.

The very different transcript levels seen for CPI-4 in two independent leaf samples from Robusta BP409 noted earlier suggested that the expression of this gene could be influenced by differences in the developmental stages of the two leaf samples. The idea that CPI-4 expression could be significantly induced in some tissues was further strengthened by the fact that very high CPI-4 expression was associated with the first cotyledons of the germinated grain. Thus, we decided to explore the effect of leaf maturity on the expression of this gene, and the other coffee CPI genes, in leaves of *Coffea arabica *T2308. Samples corresponding to four different developmental stages were used; very young leaves (VYL), young leaves (YL), mature leaves (ML) and old leaves (OL). The expression results presented in Figure 6 show that CPI-4 was very strongly induced as leaves enter their mature phase (RQ_ML _= 23.79 in set 1 and 26.31 in set 2). CPI-4 transcript levels then fell as the leaves aged further. As previously observed in *Coffea canephora *BP409 (Figure 5), very low transcript levels are seen for the CPI-1 gene in mature leaves, and this is also seen for all the other stages examined in this new experiment (Figure 6). While few transcripts were detected for CPI-2 and CPI-3 in the two early leaf stages studied here, a slightly variable, but still quite low expression was seen for CPI-2 and CPI-3 genes in the remaining two leaf stages. This latter result may indicate some slight inducible capability for the CPI-2 and CPI-3 genes, and thus it will be interesting in the future to re-examine the expression of these two CPI genes in leaf tissues subjected to different stresses (environmental and insect/fungal/bacterial attack).

## Discussion

Cysteine proteases and their inhibitors have been studied in detail for many plants, but, to date, little work has been done on these genes or proteins from coffee. Here we describe full length cDNA representing two cysteine proteinase genes, called CcCP1 and CcCP4, which show relatively exclusive expression during grain maturation and germination. We also present the characterization of full length cDNA representing four cysteine proteinase inhibitor genes (CPI-1--CPI-4) and describe the quantitative expression of these genes in coffee.

Sequence comparisons indicated that the two Robusta cysteine proteinases described represent different CP proteinases; Blast analysis against the protein database indicates that CP1 is very closely related to a papain type CP called VsCPR4 from *Vicia sativa *(GenBank Accession CAB16316), as well as a putative CP of Arabidopsis (AT3G54940, GenBank Accession NP_567010). Examination of Figure 1A shows the protein sequence of CcCP1 and its putative homologues contain a partial ERFNIN box (ERFNAQ) within an N-terminal cathepsin propeptide inhibitor domain, indicating that these polypeptides fall within the CP subfamily having an I29 domain that is found at the N-terminus of some C1 peptidases, like Cathepsin L, where it acts as a propeptide. (http://merops.sanger.ac.uk[2]; http://www.ncbi.nlm.nih.gov/Structure/cdd/cdd.shtml[32]). The I29 domain of CcCP1 and its putative homologues are located just upstream of a clear peptidase C1 superfamily domain which have strong homologies with peptidase C1A_cathepsins_B/C/X. Several other CP specific elements are also completely conserved in the three highly similar protein sequences presented in Figure 1A. The CPR4 polypeptide of *Vicia sativa *has been shown to be expressed during seed maturation and during the early part of seedling germination/growth in both the embryonic axis and the cotyledons [29]. Although the functional activity of this protein has not been proven in a recombinant form, these authors nevertheless implied the processed, and thus presumably activated polypeptide, was involved in seed storage protein mobilization. Microarray expression analysis of the potential Arabidopsis CP1 homologue (AT3G54940) indicates this gene has significant expression in developing endosperm and in the embryos of developing and germinating seedlings (https://www.genevestigator.com[33]), supporting the idea that these highly related polypeptides play a role in storage protein modification and/or mobilization. Little expression was seen for the Arabidopsis gene in other tissues under normal conditions. The expression profile of CcCP1 transcripts (Figure 2) mirrors the expression of the candidate homologues VsCPR4 and AT3G54940, ie, CcCP1 is expressed in the later stages of grain development and during germination, and implying that CcCP1 performs a similar function as the putative homologue of the other two plants. To date, it has apparently not been possible to express and/or correctly activate any of the recombinant CcCP1 homologues in order to confirm their function. The most likely explanation for this inability to verify the activity of these proteins is that the precise conditions needed to process/activate these proteins have not yet been identified.

The alignment of CcCP4 with two of the closest well characterized plant sequences (NtCP56, and SlCysEP) indicates that CcCP4 and its proposed homologues have a clear ERFNI/VN box within an N-terminal I29 type propeptide domain, followed by a peptidase C1 superfamily domain containing conserved cysteine proteinase specific sequence elements (see Figure 1B for details). All three polypeptides contain N-terminal signal sequences, and the two homologues have a C-terminal endoplasmic reticulum retention sequence (KDEL). Interestingly, the CcCP4 cDNA sequence characterized contains the C-terminal sequence KDDL. In order to explore whether the KDDL sequence was unusual in coffee, we examined the sequences of other cDNA in the coffee unigene (SGN-U613447). This unigene, which is the only clear hit obtained when the *Coffea canephora *Unigene set at http://solgenomics.net is blasted with the CcCP4 protein sequence, has 22 ESTs. Analysis of these ESTs showed that 14 have sequence data for the C-terminal end, and, interestingly 2 of these end with the KDEL sequence. This observation suggests that Robusta may have both KDDL and KDEL alleles of CcCP4. A preliminary PCR analysis of genomic fragments from this region of the CcCP4 gene in *Coffea eugenoides *and *Coffea arabica *suggests that the KDEL allele is more prominent in these species (data not shown). The significance of CP4 alleles with a C-terminal KDDL sequence is currently unclear. However, the fact that several other plant sequences in the protein database related to CcCP4 also have C-terminal KDEL or RDEL sequences (data not shown) suggests this is the more prominent form found in plants. Also, several groups have proposed that unprocessed CP-KDEL proteins are retained in the endoplasmic reticulum (ER) after synthesis, and are only processed/transported further upon specific signaling [34-36], and another group suggested that C-terminal KDDL proteins could be poorly retained in the ER [37]. These observations raise the possibility that an expressed CcCP4-KDDL protein might be poorly retained in the ER and thus could exist in unintended compartments of developing coffee grain cells, with unknown consequences. Future experiments comparing physiological or other differences between seeds of Robusta trees homozygous for the CcCP4-KDDL or CcCP4-KDEL genes could be illuminating.

Both the proposed tobacco and tomato CcCP4 homologues are known to be involved in pollen development. Zhang et al. [4] confirmed that the tobacco protein (NtCP56) encoded a functional, acid activated CP proteinase and then went on to show that anti-sense suppression of this gene can disrupt normal pollen development and cause male sterility. The tomato SlCysEP gene product was also shown to encode an acid activated CP proteinase and to be an important component of the tomato ricinosome, which is a subcellular structure believed to orchestrate the final processing/recycling of cellular proteins during plant programmed cell death [5]. In each case, the recombinant CP polypeptides produced in *E. coli *were insoluble, and, as shown here for the coffee CcCP4, needed to be refolded to demonstrate auto-cleavage and cysteine protease activity. Analysis of SlCysEP transcripts showed that they could be detected in flowers at a specific period, and that this expression was primarily limited to the stamens [5]. While the expression of NtCP56 or SlCysEP was not studied in seeds, our examination of ESTs encoding SlCysEP (http://solgenomics.net) confirmed that cDNA representing this gene can be found in *Solanum lycopersicum *EST banks from fruit, seeds, young leaves, as well as flowers (with seed libraries having the highest number of ESTs). Tomato database analysis indicates the SlCysEP gene has three introns, and that two other highly related KDEL containing "unigene" sequences can be found which potentially represent other members of this specific CP gene family. Three potential CcCP4 homologues were also identified in the Arabidopsis genome (AT5G50260, AT3G48350, and AT3G48340). Examination of the expression patterns for these genes using microarray data [33] showed that AT5G50260 expression was limited to seeds, silique and stamen/anther, although lower levels could also be found in roots, but not in stems, from plants subjected to osmotic stress. Interestingly, some induction of AT5G50260 also appeared in nematode infested roots. No significant expression was seen for this gene in other tissues; in contrast, low levels of expression were found for the Arabidopsis CcCP4 like AT3G48350 gene in many tissues, suggesting this gene may play a more general role in plant cells. The only two situations that appeared to increase AT3G48350 transcript levels were treatment with uv and dramatic changes in light conditions (dark/light shifts). No probe sets were identified for the gene sequence AT3G48340, so the expression of this gene is not known.

Overall, the CcCP4 expression data presented are consistent with our proposal that CcCP4 may be involved in the PCD associated with coffee grain germination and post-germination stages. Although no significant CcCP4 expression was detected in the Robusta BP409 flower sample tested (data not shown), this may be due to the limited developmental time frame we analysed. New analysis, using several different stages of flower development is clearly needed to clarify the expected participation of CcCP4 in coffee pollen development. It is interesting to note the *Vicia sativa *Proteinase A (CcCP4 homologue) was not detected during vetch seed development, but was detected in the cotyledons in the later stages of germination and post-germination (was also not detected in the seedling axis) [29,38]. These observations, together with the fact that purified *Vicia sativa *Proteinase A was capable of completely digesting the vetch storage proteins vicilin and legumin, led the authors to propose that Proteinase A was not involved in seed development or in the early part of storage protein mobilization, but was important for later stages of germination which involved much more extensive proteolysis [38]. This contrasts with coffee where there appears to be two periods of CcCP4 expression, one in the developing grain (which may continue into the first part of germination), and another new burst of transcription beginning around the T2 stage of germination up to T5 stage. We currently do not know the significance of finding low CcCP4 expression in the developing grain, although we do note that the "absence" of Protein A in *Vicia sativa *in developing seeds [38] could be due to the less sensitive detection method used earlier (northern blotting versus QRT-PCR here).

The quantitative expression analysis of the four CPI genes (Figure 5) showed that CcCPI-2 and CcCPI-3 are expressed in most tissues and that their levels of expression do not vary broadly. In contrast, CPI-1 had increasingly higher expression as grain development progresses (> 100 fold increase from immature to mature stages) and also showed relatively strong expression during the T2 to T5 stages of grain germination and post germination. Little CcCPI-1 expression was detected in the other tissues tested. For CcCPI-4, extremely high levels of transcripts were seen exclusively in the T5 and T6 stages of post-germination, corresponding to stages in which the cotyledons are forming. The significance of this observation is not known, but one interesting line of future investigation will be to determine whether CPI-4 expression contributes to insect tolerance/resistance at this delicate stage of plantlet development. By examining gene expression at different stages of leaf development, we also found that while CcCPI-4 is weakly expressed in young leaves, its expression increases dramatically in mature leaves (Figure 6). No significant expression of CcCPI-4 was found for the other tissues tested, except a low level in the roots from Robusta BP409 cDNA set used in Figure 5 (RQ = 0.53), which raises the interesting possibility that the higher levels of one or more CcCPI proteins could reduce damage by root pests like nematodes. Overall, the coffee CPI gene expression data suggest that CPI-2 and CPI-3 could be CP inhibitors with mostly "house-keeping" functions, while CPI-1 may play an important role during grain development, and CPI-4 could contribute to reducing damage by insects during the early life of the plantlets (first cotyledons), and perhaps in mature/old leaves and roots. Finally, as the peptide/amino acid profile of a coffee has an important impact on flavour and aroma generation during coffee grain roasting [24,39], further research is warranted to investigate possible links that may exist between the allelic variation in genes encoding coffee cysteine proteinases and cysteine proteinase inhibitors and the flavour/aroma quality associated with the grain of different coffee varieties.

## Conclusions

Several cysteine proteinase and cysteine proteinase inhibitor genes with strong, relatively specific expression during coffee grain maturation and germination are presented. The temporal expression of the CcCP1 gene suggests it is involved in modifying proteins during late grain maturation and germination. The expression pattern of CcCP4, and its close identity with KDEL containing CP proteins, implies this proteinase may play a role in protein and/or cell remodelling during late grain germination, and that it is likely to play a strong role in the programmed cell death associated with post-germination of the coffee grain. Expression analysis of the cysteine proteinase inhibitor genes suggests that CcCPI-1 could primarily be involved in modulating the activity of grain CP activity; while CcCPI-4 may play roles modulating grain CP activity and in the protection of the young coffee seedlings from insects and pathogens. CcCPI-2 and CcCPI-3, having lower and more widespread expression, could be more general "house-keeping" CPI genes. The data generated opens up new avenues to explore the potential contribution of proteinases to coffee quality and facilitates new research to investigate the possibility that coffee cysteine proteinase inhibitors may help reduce damage caused by some plant pests.

## Abbreviations

CP: Cysteine proteinase; CPI: Cysteine proteinase inhibitor; QRT-PCR: Quantitative reverse transcriptase-PCR; PCD: Programmed cell death; VCR: Vacuolar receptor; PSV: Protein storage vesicles; DAI: Days after imbibition; IBS: Inclusion bodies; ER: Endoplasmic reticulum

## Authors' contributions

JMcC, ML and MBA were involved in the design, supervision and interpretation of the experiments, and all of the authors contributed to the analysis of the data. JMcC conceived the work and drafted the manuscript. MBA, GC and VC carried out the initial characterization and expression analysis of the gene sequences. ML and NM produced and characterized the recombinant CP1 and CP4 proteins. The quantitative transcript analysis and additional sequence analysis of these genes was done by ML, NM and VC All the authors have read and approved the manuscript.

## GenBank accession numbers

*Coffea canephora *protein sequence data associated with this work article has been deposited in GenBank under following accession numbers: CcCPI-1 (AEQ54766), CcCPI-2 (AEQ54767), CcCPI-3 (AEQ54768), CcCPI-4 (AEQ54769), CcCP1 (AEQ54770), CcCP4 (KDDL-tailed) (AEQ54771) and CcCP4 (partial--KDEL-tailed) (AEQ54772).

## Supplementary Material

Additional file 1 **Robusta BP409 germination samples used for QRT-PCR**. The times of sampling are given for each stage. The "T1" sample is the sterilized and washed material obtained just before placing on the "germination" media. The "T4" sample (14 Days) showed the "First Evidence" of germination, ie. the radical has just started to protrude from the grain. In T1-T4, all the sample shown was used for RNA extraction. For T5 and T6 only the grain and first cotyledons (and remaining grain material) respectively were used to make RNA. Click here for file

Additional file 2 **HIS-SUMO-CcCP4 recombinant protein purification steps**. Samples from the different purification steps were run on a 8-6% SDS-polyacrylamide gel and stained with Coomassie. Starting material was an induced BL21 (DE3) + pET-SUMO/CP4 whole cell lysate. Arrow indicates HIS-SUMO-CcCP4. **Panel A: Lane M**, Molecular marker proteins with the sizes shown on the left in kDa. **Lane 1**, Pellet of induced whole cell lysate (material insoluble in the extraction Buffer); **Lane 2**, Washed Inclusion bodies; **Lane 3-Lane 5**, Successive flow-through fractions collected during His-Tag column washes; **Lane 6**, First eluate fraction of the His-Tag column; **Lane 7**, Pool of second and third His-Tag column eluates before dialysis step. **Panel B: Lane M**, Molecular marker proteins. **Lane 1**, Purified recombinant HIS-SUMO-CP4 protease of pooled second and third eluates after dialysis step. Click here for file

Additional file 3 **Aurto-catalytic processing/activation of recombinant HIS-SUMO-CP4 proteinase**. 10 μL (3.2 μg) of His-Tag column purified and dialysed recombinant HIS-SUMO-CP4 was added to 20 μL acid buffer (sodium formate 50 mM pH3), then either A) immediately stopped by the addition of 14 μl 5x loading buffer (Lane 1, T = 0), or B) incubated in a water-bath at 37°C for 30 sec (Lane 2, T = 30 sec) or 1 h (Lane 3, T = 1 h) followed by adding 14 μl 5x loading buffer to stop the reactions. The three samples were then heated at 95°C for 7 min and run on an 8-16% SDS-PAGE gel followed by silver staining with the SilverSNAP Stain Kit II (ThermoScientific). Arrow indicates processed, activated CP4 proteinase. The calculated size of the full length HIS-SUMO-CP4 was 59.9 kDa (its predicted size is 50.7 kDa), while the size of the processed, active CP4 indicated by the arrow was calculated to be 32.6 kDa (which is close to the 25.2 kDa size predicted if HIS-SUMO-CP4 is cleaved in a similar position to the that seen for *Nicotiana tabacum *NtCP56 recombinant protein (Zhang et al. ref [4]). Click here for file

Additional file 4 **Effect of cysteine protease inhibitor E-64C on CcCP4 activity**. The inhibitory effect of the cysteine protease inhibitor E-64C on the activity of CcCP4 was tested as follows: 10 μL (3.2 μg) His-tag purified and dialysed recombinant HIS-SUMO-CP4 protease was added to 20 μl sodium formate (pH3) and incubated 30 sec in a 37°C water-bath. Then, either 100 μM E-64C (Panel A), or 10 μM E-64C (Panel B), or 1 μM E-64C (Panel C), or no E-64C (Panel D, control), were added, immediately followed by the addition of 6.7 μL BSA reaction buffer (see methods section). For each reaction, 3 μl samples were taken at the start (T = 0), and at T = 5 min, T = 10 min, T = 3 h, T = 4 h30, and immediately added to 5 ul 5x SDS gel loading buffer. The samples were subsequently run on 8-16% SDS-PAGE gels and stained by coomassie. **Lane M**, Molecular marker with the sizes shown on the left in kDa (Biorad Precision Protein™ Standards, prestained). Click here for file

Additional file 5 **Plasmid names are given for all the cDNA described in the manuscript**. The sizes of the plasmid inserts and length of encoded proteins are also given. Click here for file
