# Supplementary material for: Coffee cysteine proteinases and related inhibitors with high expression during grain maturation and germination
Source: BMC Plant Biol. 2012 Mar 1;12:31. doi: 10.1186/1471-2229-12-31 (PMC3311568; doi:10.1186/1471-2229-12-31)
Supplement: Additional file 1 — Robusta BP409 germination samples used for QRT-PCR. The times of sampling are given for each stage. The "T1" sample is the sterilized and washed material obtained just before placing on the "germination" media. The "T4" sample (14 Days) showed the "First Evidence" of germination, ie. the radical has just started to protrude from the grain. In T1-T4, all the sample shown was used for RNA extraction. For T5 and T6 only the grain and first cotyledons (and remaining grain material) respectively were used to make RNA. [file 1471-2229-12-31-S1.PPTX]

## Slide 1
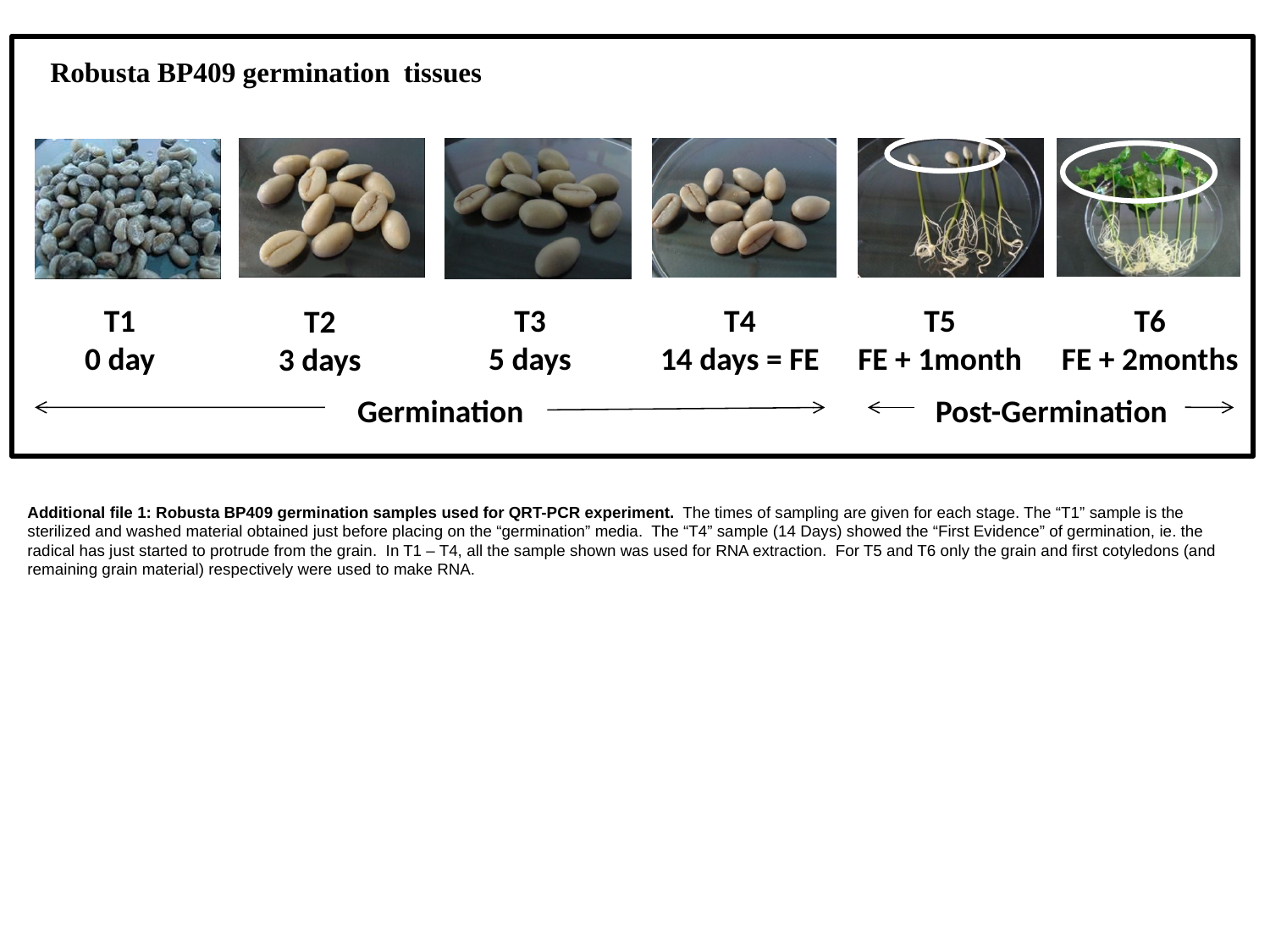

Robusta BP409 germination tissues
T1
0 day
T3
5 days
T4
14 days = FE
T5
FE + 1month
T6
FE + 2months
T2
3 days
Post-Germination
Germination
Additional file 1: Robusta BP409 germination samples used for QRT-PCR experiment. The times of sampling are given for each stage. The “T1” sample is the sterilized and washed material obtained just before placing on the “germination” media. The “T4” sample (14 Days) showed the “First Evidence” of germination, ie. the radical has just started to protrude from the grain. In T1 – T4, all the sample shown was used for RNA extraction. For T5 and T6 only the grain and first cotyledons (and remaining grain material) respectively were used to make RNA.
