# Supplementary material for: Coffee cysteine proteinases and related inhibitors with high expression during grain maturation and germination
Source: BMC Plant Biol. 2012 Mar 1;12:31. doi: 10.1186/1471-2229-12-31 (PMC3311568; doi:10.1186/1471-2229-12-31)
Supplement: Additional file 2 — HIS-SUMO-CcCP4 recombinant protein purification steps. Samples from the different purification steps were run on a 8-6% SDS-polyacrylamide gel and stained with Coomassie. Starting material was an induced BL21 (DE3) + pET-SUMO/CP4 whole cell lysate. Arrow indicates HIS-SUMO-CcCP4. Panel A: Lane M, Molecular marker proteins with the sizes shown on the left in kDa. Lane 1, Pellet of induced whole cell lysate (material insoluble in the extraction Buffer); Lane 2, Washed Inclusion bodies; Lane 3-Lane 5, Successive flow-through fractions collected during His-Tag column washes; Lane 6, First eluate fraction of the His-Tag column; Lane 7, Pool of second and third His-Tag column eluates before dialysis step. Panel B: Lane M, Molecular marker proteins. Lane 1, Purified recombinant HIS-SUMO-CP4 protease of pooled second and third eluates after dialysis step. [file 1471-2229-12-31-S2.PPTX]

## Slide 1
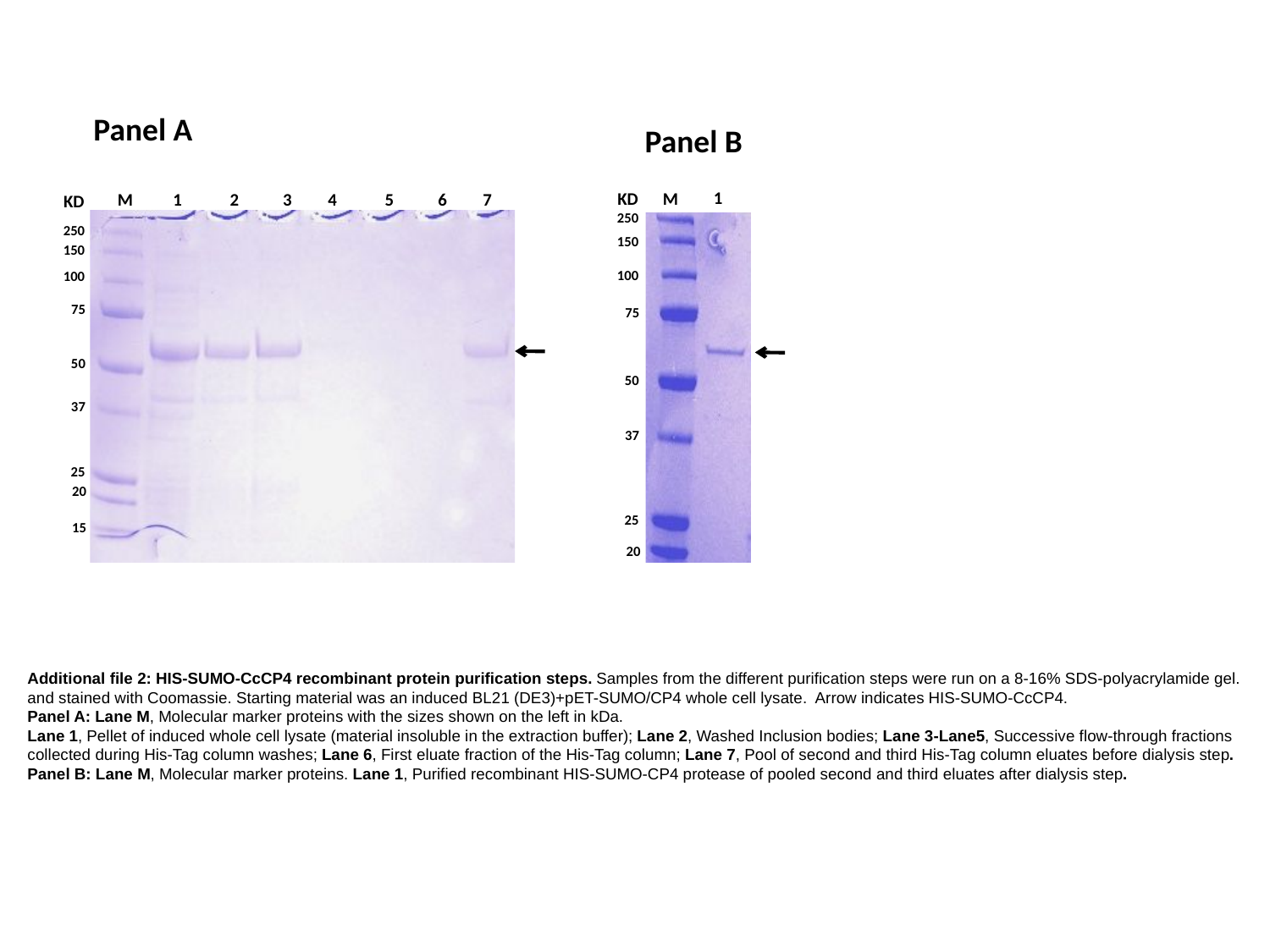

Panel A
Panel B
1
M
KD
M 1 2 3 4 5 6 7
KD
250
250
150
150
100
100
75
75
50
50
37
37
25
20
25
15
20
Additional file 2: HIS-SUMO-CcCP4 recombinant protein purification steps. Samples from the different purification steps were run on a 8-16% SDS-polyacrylamide gel. and stained with Coomassie. Starting material was an induced BL21 (DE3)+pET-SUMO/CP4 whole cell lysate. Arrow indicates HIS-SUMO-CcCP4.
Panel A: Lane M, Molecular marker proteins with the sizes shown on the left in kDa.
Lane 1, Pellet of induced whole cell lysate (material insoluble in the extraction buffer); Lane 2, Washed Inclusion bodies; Lane 3-Lane5, Successive flow-through fractions collected during His-Tag column washes; Lane 6, First eluate fraction of the His-Tag column; Lane 7, Pool of second and third His-Tag column eluates before dialysis step.
Panel B: Lane M, Molecular marker proteins. Lane 1, Purified recombinant HIS-SUMO-CP4 protease of pooled second and third eluates after dialysis step.
