# Supplementary material for: Coffee cysteine proteinases and related inhibitors with high expression during grain maturation and germination
Source: BMC Plant Biol. 2012 Mar 1;12:31. doi: 10.1186/1471-2229-12-31 (PMC3311568; doi:10.1186/1471-2229-12-31)
Supplement: Additional file 3 — Aurto-catalytic processing/activation of recombinant HIS-SUMO-CP4 proteinase. 10 μL (3.2 μg) of His-Tag column purified and dialysed recombinant HIS-SUMO-CP4 was added to 20 μL acid buffer (sodium formate 50 mM pH3), then either A) immediately stopped by the addition of 14 μl 5x loading buffer (Lane 1, T = 0), or B) incubated in a water-bath at 37°C for 30 sec (Lane 2, T = 30 sec) or 1 h (Lane 3, T = 1 h) followed by adding 14 μl 5x loading buffer to stop the reactions. The three samples were then heated at 95°C for 7 min and run on an 8-16% SDS-PAGE gel followed by silver staining with the SilverSNAP Stain Kit II (ThermoScientific). Arrow indicates processed, activated CP4 proteinase. The calculated size of the full length HIS-SUMO-CP4 was 59.9 kDa (its predicted size is 50.7 kDa), while the size of the processed, active CP4 indicated by the arrow was calculated to be 32.6 kDa (which is close to the 25.2 kDa size predicted if HIS-SUMO-CP4 is cleaved in a similar position to the that seen for Nicotiana tabacum NtCP56 recombinant protein (Zhang et al. ref [4]). [file 1471-2229-12-31-S3.PPTX]

## Slide 1
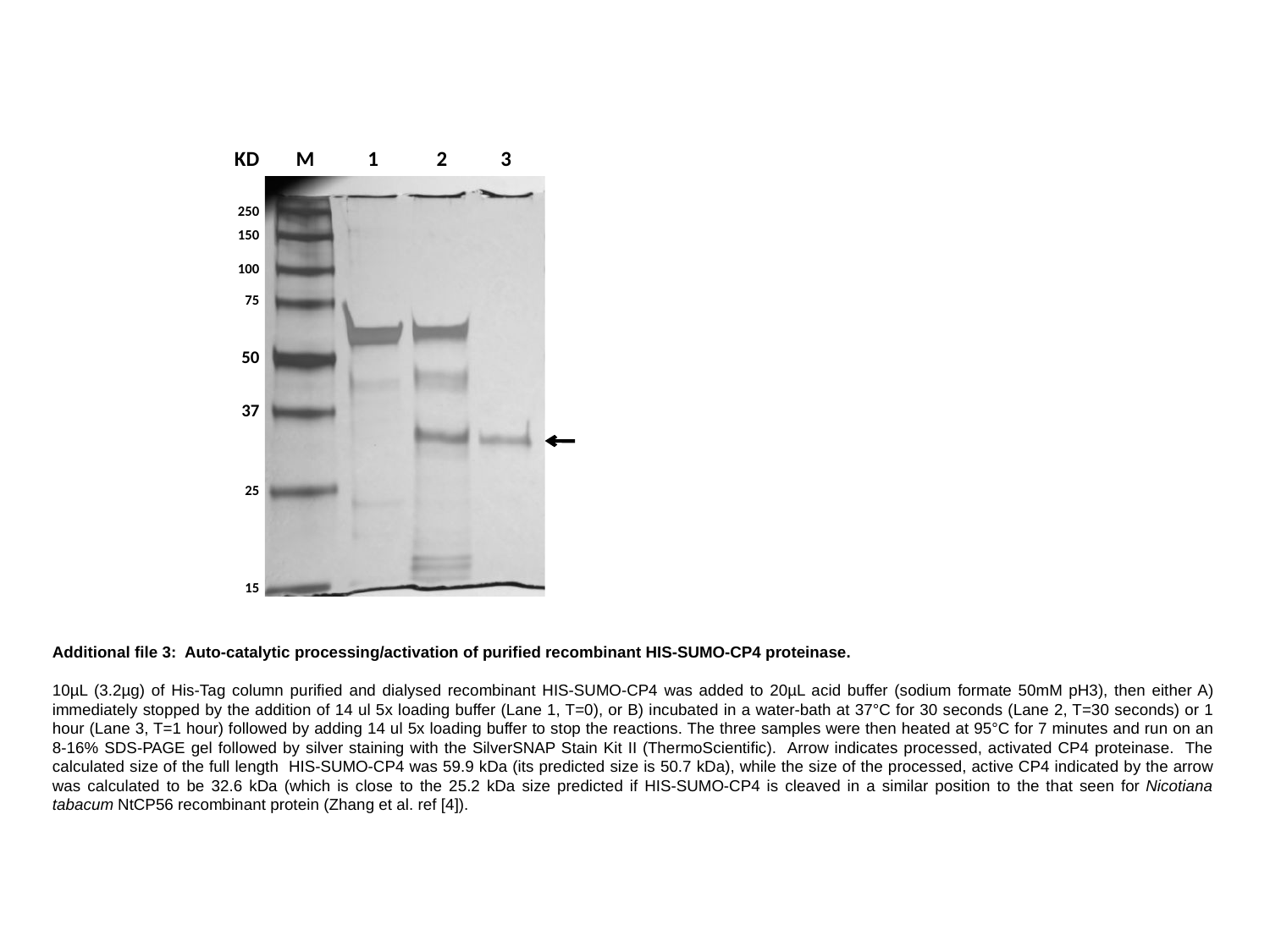

KD
250
 150
100
75
50
37
25
15
 M 1 2 3
Additional file 3: Auto-catalytic processing/activation of purified recombinant HIS-SUMO-CP4 proteinase.
10µL (3.2µg) of His-Tag column purified and dialysed recombinant HIS-SUMO-CP4 was added to 20µL acid buffer (sodium formate 50mM pH3), then either A) immediately stopped by the addition of 14 ul 5x loading buffer (Lane 1, T=0), or B) incubated in a water-bath at 37°C for 30 seconds (Lane 2, T=30 seconds) or 1 hour (Lane 3, T=1 hour) followed by adding 14 ul 5x loading buffer to stop the reactions. The three samples were then heated at 95°C for 7 minutes and run on an 8-16% SDS-PAGE gel followed by silver staining with the SilverSNAP Stain Kit II (ThermoScientific). Arrow indicates processed, activated CP4 proteinase. The calculated size of the full length HIS-SUMO-CP4 was 59.9 kDa (its predicted size is 50.7 kDa), while the size of the processed, active CP4 indicated by the arrow was calculated to be 32.6 kDa (which is close to the 25.2 kDa size predicted if HIS-SUMO-CP4 is cleaved in a similar position to the that seen for Nicotiana tabacum NtCP56 recombinant protein (Zhang et al. ref [4]).
