# Supplementary material for: Coffee cysteine proteinases and related inhibitors with high expression during grain maturation and germination
Source: BMC Plant Biol. 2012 Mar 1;12:31. doi: 10.1186/1471-2229-12-31 (PMC3311568; doi:10.1186/1471-2229-12-31)
Supplement: Additional file 4 — Effect of cysteine protease inhibitor E-64C on CcCP4 activity. The inhibitory effect of the cysteine protease inhibitor E-64C on the activity of CcCP4 was tested as follows: 10 μL (3.2 μg) His-tag purified and dialysed recombinant HIS-SUMO-CP4 protease was added to 20 μl sodium formate (pH3) and incubated 30 sec in a 37°C water-bath. Then, either 100 μM E-64C (Panel A), or 10 μM E-64C (Panel B), or 1 μM E-64C (Panel C), or no E-64C (Panel D, control), were added, immediately followed by the addition of 6.7 μL BSA reaction buffer (see methods section). For each reaction, 3 μl samples were taken at the start (T = 0), and at T = 5 min, T = 10 min, T = 3 h, T = 4 h30, and immediately added to 5 ul 5x SDS gel loading buffer. The samples were subsequently run on 8-16% SDS-PAGE gels and stained by coomassie. Lane M, Molecular marker with the sizes shown on the left in kDa (Biorad Precision Protein™ Standards, prestained). [file 1471-2229-12-31-S4.PPTX]

## Slide 1
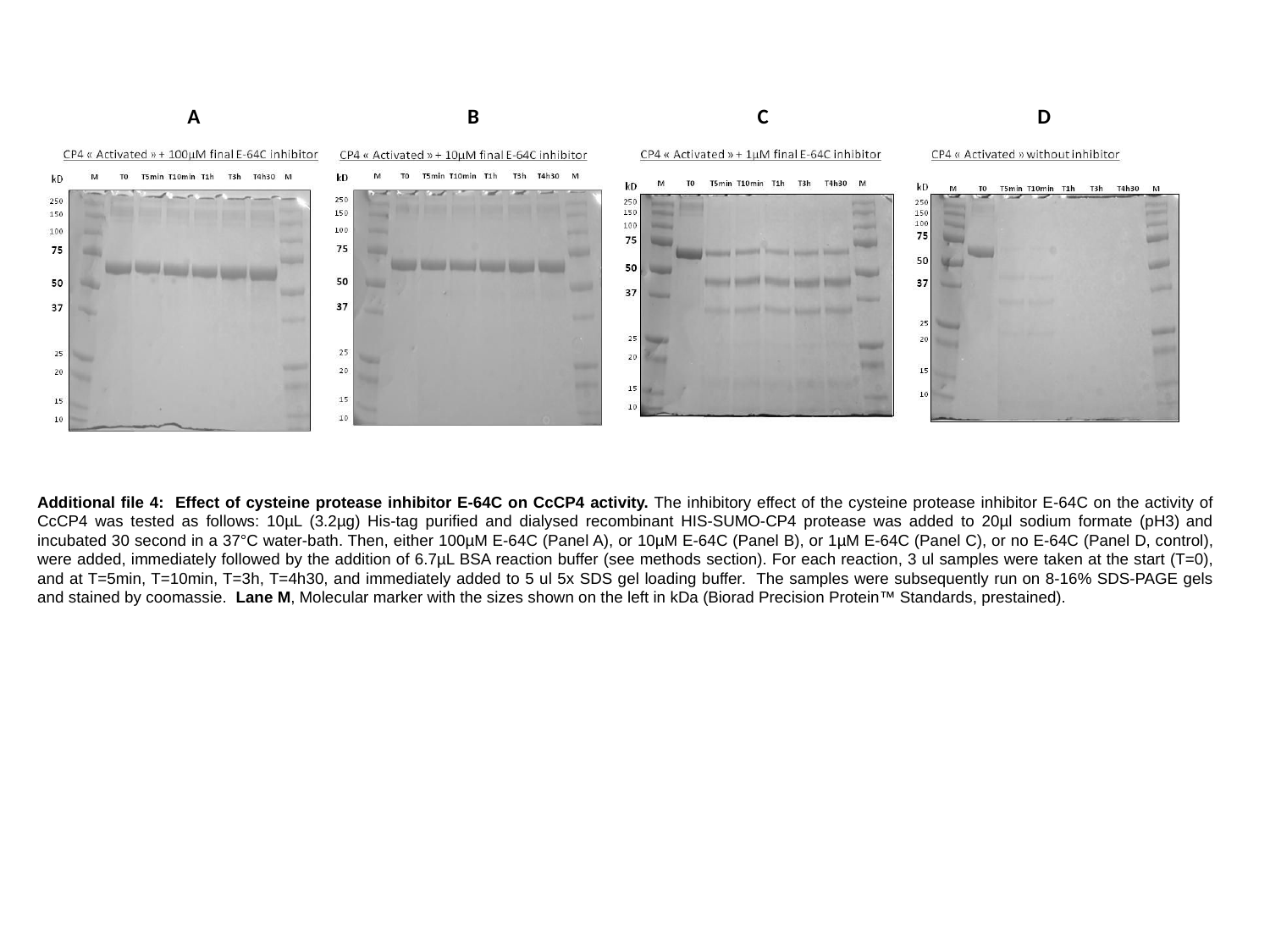

A
B
C
D
Additional file 4: Effect of cysteine protease inhibitor E-64C on CcCP4 activity. The inhibitory effect of the cysteine protease inhibitor E-64C on the activity of CcCP4 was tested as follows: 10µL (3.2µg) His-tag purified and dialysed recombinant HIS-SUMO-CP4 protease was added to 20µl sodium formate (pH3) and incubated 30 second in a 37°C water-bath. Then, either 100µM E-64C (Panel A), or 10µM E-64C (Panel B), or 1µM E-64C (Panel C), or no E-64C (Panel D, control), were added, immediately followed by the addition of 6.7µL BSA reaction buffer (see methods section). For each reaction, 3 ul samples were taken at the start (T=0), and at T=5min, T=10min, T=3h, T=4h30, and immediately added to 5 ul 5x SDS gel loading buffer. The samples were subsequently run on 8-16% SDS-PAGE gels and stained by coomassie. Lane M, Molecular marker with the sizes shown on the left in kDa (Biorad Precision Protein™ Standards, prestained).
