# Supplementary material for: Coffee cysteine proteinases and related inhibitors with high expression during grain maturation and germination
Source: BMC Plant Biol. 2012 Mar 1;12:31. doi: 10.1186/1471-2229-12-31 (PMC3311568; doi:10.1186/1471-2229-12-31)
Supplement: Additional file 5 — Plasmid names are given for all the cDNA described in the manuscript. The sizes of the plasmid inserts and length of encoded proteins are also given. [file 1471-2229-12-31-S5.PPTX]

## Slide 1
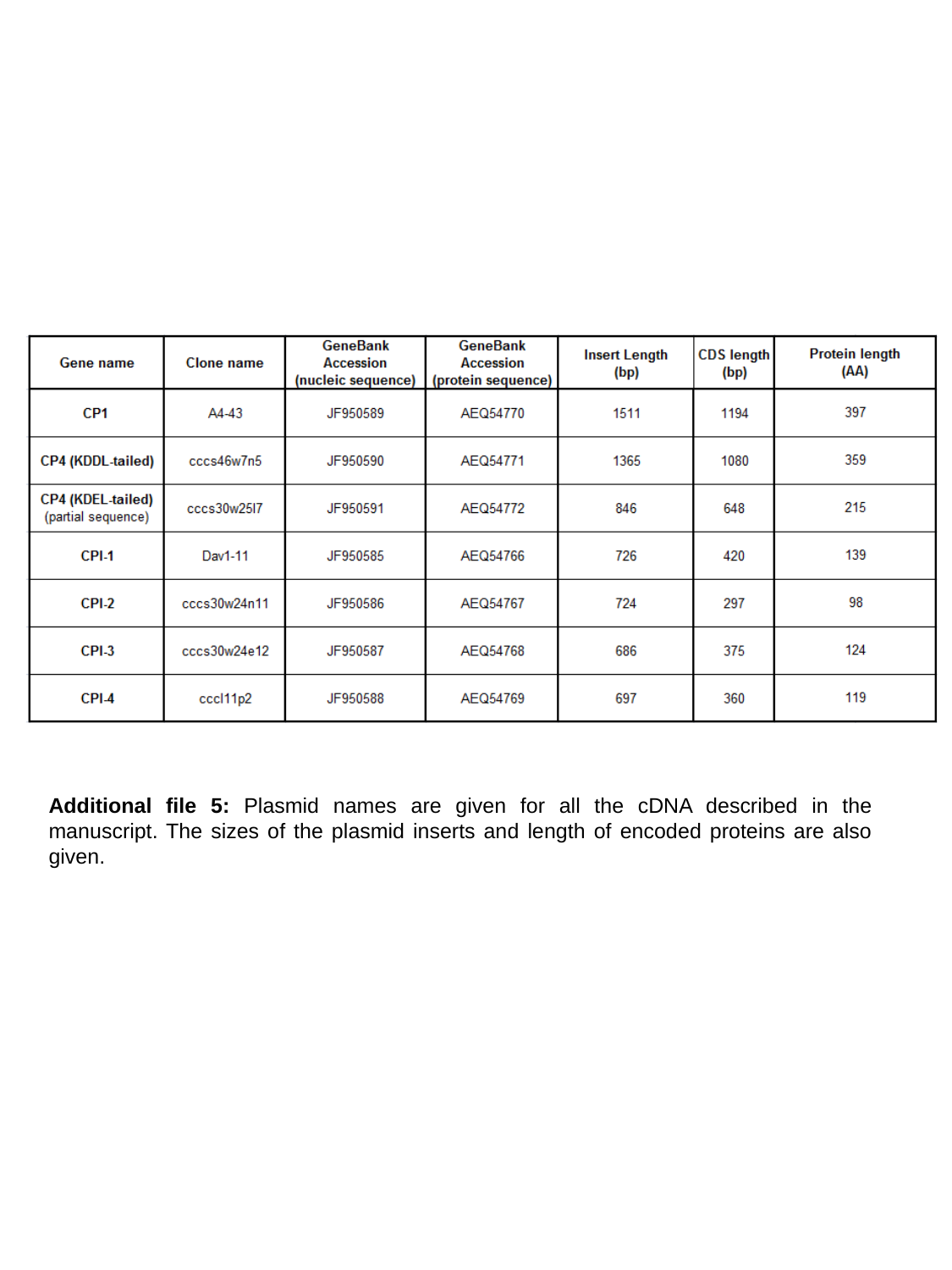

Additional file 5: Plasmid names are given for all the cDNA described in the manuscript. The sizes of the plasmid inserts and length of encoded proteins are also given.
